# Distress that penetrates the skin: the impact of intersectional violence on black female nurses’ mental health

**DOI:** 10.1590/0034-7167-2025-0263

**Published:** 2026-06-12

**Authors:** Isabela de Freitas Bahia Pereira, Tiago Braga do Espírito Santo, Cecília Maria Izidoro Pinto, Eliane Oliveira Andrade Paquiela, Eluana Borges Leitão de Figueiredo, Ricardo de Mattos Russo Rafael, Caroline Silva Batista Alves, Karoline Nascimento Souza

**Affiliations:** I Universidade do Estado do Rio de Janeiro. Rio de Janeiro, Rio de Janeiro, Brazil; II Universidade Federal do Rio de Janeiro. Rio de Janeiro, Rio de Janeiro, Brazil

**Keywords:** Intersectional Framework, Mental Health, Nurses, Women, Racism., Marco Interseccional, Salud Mental, Enfermería, Mujeres, Racismo.

## Abstract

**Objectives:**

to analyze the experiences of black female nurses at a university hospital in Rio de Janeiro from an intersectional perspective, with an emphasis on their psychological distress.

**Methods:**

a qualitative study with 12 participants, using narrative interviews and Bardin’s content analysis.

**Results:**

data analysis revealed three categories. In this study, we highlight the second, “Distress that penetrates the skin: the impact of intersectional violence on mental health”, which highlights emotional demands, work overload, experiences of prejudice, and recurring professional devaluation. Psychological distress results from intersectional oppressions in healthcare institutions, affecting well-being and quality of care. The theoretical contributions of Lélia Gonzalez, Sueli Carneiro, and Kimberlé Crenshaw allow us to understand how these oppressions intertwine in these professionals’ daily lives.

**Final Considerations:**

intersectional violence acts in a coordinated manner, producing exclusions and psychological distress, which reinforces the urgency for anti-racist and gender-equal institutional policies.

## RESUMEN

**Objetivos:**

analizar las experiencias de enfermeras negras en hospital universitario de Río de Janeiro desde una perspectiva interseccional, con énfasis en su sufrimiento psicológico.

**Métodos:**

estudio cualitativo con 12 participantes, mediante entrevistas narrativas y análisis de contenido de Bardin.

**Resultados:**

el análisis de datos reveló tres categorías. En este estudio, destacamos “Sufrimiento que penetra la piel: el impacto de la violencia interseccional en la salud mental”, que destaca las exigencias emocionales, sobrecarga laboral, experiencias de prejuicio y devaluación profesional recurrente. El sufrimiento psicológico es resultado de las opresiones interseccionales en instituciones de salud, lo que afecta el bienestar y la calidad de la atención. Las contribuciones teóricas de Lélia Gonzalez, Sueli Carneiro y Kimberlé Crenshaw nos permiten comprender cómo estas opresiones se entrelazan en la vida cotidiana de estos profesionales.

**Consideraciones Finales:**

la violencia interseccional actúa de forma coordinada, generando exclusiones y sufrimiento psicológico, lo que refuerza la urgencia de políticas institucionales antirracistas y de igualdad de género.

## INTRODUCTION

The debate on gender inequalities and the harmful effects of racism has increasingly gained traction on nursing research and work agendas. However, they are generally treated as isolated forms of oppression, an aspect that, while important, disregards the intersecting effects of these systems of oppression and violence on bodies. In an attempt to bridge this gap, the concept of intersectionality, as formulated by Kimberlé Crenshaw^(1)^ and later developed by Patricia Hill Collins^(2)^, provides a fundamental theoretical framework for analyzing how several systems of oppression operate simultaneously and interrelatedly.

Kimberlé Crenshaw^(1)^ argues that intersectionality goes beyond the simple sum of oppressions, constituting a paradigm that highlights how different axes of power intersect, producing unique experiences of discrimination. Patricia Hill Collins and Sirma Bilge^(2)^ complement this perspective by emphasizing that intersectionality is both an analytical tool and a critical practice that examines how power relations are maintained and reproduced through the interaction between social structures, cultural representations, and identity processes. This theoretical development enables a deeper understanding of the social inequalities that affect groups historically excluded from society^(2)^.

By combining two or more categories that allow us to understand the production of the subject, such as social class, gender, race/ethnicity, sexuality, among others, the concept brings as its basic idea the possibility of explaining how norms, values, ideologies, and discourses influence social structures and identity formation. Furthermore, it allows us to deepen our understanding of how identity formation, which, by their theoretical nature, are established based on differences, affect the formation of individuals’ subjectivities^(3)^. In this regard, intersectionality, as an analytical tool for understanding psychological distress, it allows us to understand how social markers of difference are associated with and are responsible for structuring lives through injustices and inequalities.

The concept of race is linked to historical circumstances. Always permeated by conflicts, power relations, and authoritarian decisions, racial relations are marked by prejudice, inequality, stereotypes, and discrimination. Racism, culturally embedded in society through conscious and unconscious practices, is objectively linked to the recurring relationship of white superiority over black people and the constant presence of the agency of whiteness in shaping these relations of power and oppression^(4)^.

Gender, in turn, constitutes an analytical and political category that highlights how sexual differences are converted into social inequalities through historical and cultural processes. Gender refers not only to individual identity, but to a system of power relations that hierarchically organize the social experiences of men and women. In the context of healthcare work, gender relations manifest themselves in the feminization of professions such as nursing, producing an unequal distribution of prestige, authority, and pay^(5)^. These relationships become more complex when intersected with race and class, determining specific forms of vulnerability and discrimination that particularly affect black women in the workplace.

Finally, social class relates to the way power is stratified in society based on their access to economic capital^(6)^. From an intersectional perspective, the aforementioned axes of power intertwine, establishing a deep intertwining of racial, gender, and class relations within the social structure, permeating the personal and collective lives of individuals and groups. These relations are simultaneously constituted as practices of identity and otherness, inclusion and exclusion, and equality and inequality.

Hence, racism, sexism, and classism cannot be understood in isolation, as they are historically intertwined with economic and social dynamics, operating as mechanisms for legitimizing exploitation and social hierarchy. From this perspective, racial, gender, and class inequalities are not distinct spheres, but rather intertwined expressions of a single historical process of domination and subordination shaped by coloniality and the development of capitalism. Thus, intersectional analysis highlights their deep roots in the social structure of individuals, groups, and classes, shaped by dynamics of diversity and inequality. These dynamics continually fuel intolerance, prejudice, and stereotypes that, although historically transformed, persist as central dilemmas of the contemporary world.

Nursing care, historically practiced by women, is a field of knowledge and practices permeated by epistemological disputes, power relations, and processes of subordination. Ehrenreich and English^(7)^ demonstrate that, from healers and midwives to professional nursing consolidation, women’s empirical and community knowledge was delegitimized by the rise of modern medicine, marked by a masculine, scientific, and institutionalized logic. In Brazil, this process was articulated with a hygienist and disciplinary project that incorporated Eurocentric and racialized standards, linking the professionalization of nursing to subordination to the medical profession and marginalizing popular care practices^(8)^.

Studies such as those by Padilha, Nelson, and Borenstein^(9)^ show that nurses’ identity in Brazil was constructed based on biographical trajectories permeated by tensions, resistance, and adaptation to hierarchical structures of care. Complementarily, Magalhães^(10)^ analyzes how gender stereotypes associated with the idea of caring for the feminine nature, docility, and selflessness have remained a structuring element of the social perception and professional appreciation of nursing. Recent reviews indicate that scientific production in the field still reproduces these narratives, although there is also a strengthening of critical discourses that challenge them^(11)^.

In the hospital context, understood as a disciplinary institution that regulates bodies, times, and knowledge^(12,13)^, nursing work oscillates between technical-relational autonomy and the subordinate execution of medical prescriptions. The literature on racial relations demonstrates that this process produces distress that is not distributed homogeneously, as social markers, such as gender, race, and class, operate as structuring axes of inequality, producing specific vulnerabilities and naturalizing hierarchies in care^(14)^.

Black nurses face constant demands that force them to demonstrate their knowledge and competence. This effort transforms daily work into a continuous challenge to prove, through qualifications, specializations, and experience, that they have qualified training and deserve recognition, even when their capabilities are evident for the positions they hold. Professional practice, therefore, becomes marked by a constant tension between skill and validation, demonstrating how structural racism is embedded in everyday care and leadership practices.

In this context, it is not difficult to understand that inequality production tends to generate distress, here understood as psychological distress, established as a complex and multifaceted phenomenon characterized by its subjective and individual nature, manifesting itself in broad and diverse ways. This distress is often associated with experiences of strong emotional impact and can be expressed through feelings such as sadness, frustration, helplessness, and a perception of incapacity. The broader understanding of mental health adopted in this research favors a comprehensive approach to the subject that transcends the mere emphasis on psychiatric diagnosis and nosological classification, focusing on the effects that personal experiences have on social and individual life. In this regard, psychological distress is conceived as a subjective expression of adverse experiences that affect individuals’ relationship with the world around them. This perspective overcomes the limitations of traditional diagnostic models by prioritizing subjectivity and unique needs as central axes of mental healthcare^(15)^.

Considering the elements presented, the need to produce knowledge about the intersections present in the existence of black nurses is evident, highlighting those who work in the hospital space. The hospital is configured as a place of great insertion for the category^(16)^, as well as a historical field of dispute between categories and hierarchies recognized socially in an uneven manner, with expansions constantly strained precisely by the oppressions produced by systems of racialization, gender inequities, and class differentiation.

Thus, the study, by giving voice and affirming these women as valid interlocutors of their lives, recognizes their spaces, valuing their experiences and experiences in their daily work. To this end, the research question is: how do intersectional axes of power influence black nurses’ mental health and psychological distress?

## OBJECTIVES

To analyze the experience of black nurses at a university hospital in Rio de Janeiro, focusing on reflection about their psychological distress.

## METHODS

### Ethical aspects

The study was conducted in accordance with national ethical guidelines, and was approved by the *Universidade do Estado do Rio de Janeiro* (UERJ) Research Ethics Committee, whose opinion is attached to this submission. Written informed consent was obtained from all individuals and signed prior to the narrative interview. Participation was voluntary, and nurses’ anonymity was guaranteed by replacing their names with designations of African origin, in a symbolic gesture of valuing ancestry and resistance, inspired by Lélia Gonzalez’s^(17)^ reflections on identity and culture.

### Study design

This is exploratory research with a qualitative approach, whose methodology seeks to understand aspects of reality that cannot be identified by numbers, based on theories to investigate social phenomena in depth. The study aims to reveal processes that are still little known, especially in relation to specific groups, enabling both the construction of new perspectives and the revision and elaboration of concepts and categories throughout the investigation^(18)^.

Exploratory research, in turn, seeks to understand and explain the causes, consequences, and characteristics of a phenomenon, providing researchers with greater insight into the topic. To achieve this, familiarity, knowledge, and understanding are necessary in the initial stages of investigation, aiding in the development or creation of explanatory hypotheses, the determination of variables, the investigation of human behavior, and the identification of concepts or variables^(19)^.

The study followed the COnsolidated Criteria for Reporting Qualitative Research (COREQ) guidelines, which establishes a set of 32 verification items to ensure methodological rigor and transparency in the conduct and reporting of qualitative research. The use of COREQ enabled a detailed and systematic description of essential methodological aspects, with an emphasis on the research team’s reflexivity, study design, and data analysis, contributing to the scientific integrity of the research^(20)^.

### Study setting

The research was carried out at a university hospital in the state of Rio de Janeiro, an institution that meets the requirements of the Ministry of Education and Culture and which, in its work routine, welcomes undergraduate nursing students, *lato sensu* and *stricto sensu* graduate students, as well as nurses and professionals from other health areas.

### Data source

The sample included black women (black and brown) over the age of 18, with a college degree in nursing, and working at the hospital at the time of data collection. Men, self-identified white women, and individuals not working in the hospital, on leave, or on vacation were excluded.

### Data collection and organization

Data collection was conducted through narrative interviews^(21)^, a method that emphasizes listening to lived experiences as essential content for intersectional analysis. The focus was on understanding participants’ experiences in hospital work settings, especially regarding psychological distress.

The interviews took place during July and August 2024, and were recorded and transcribed. The research team provided a setting in which participants felt comfortable sharing their experiences and recounting their feelings, obtaining narrative interviews lasting an average of 40 minutes.

For data collection, we adopted the snowball strategy, a non-probabilistic approach widely used to access hard-to-reach populations, such as black nurses, who are often overlooked in research contexts. This technique is based on the construction of referral chains, in which initial participants (seeds), selected by convenience, are invited to recommend other individuals belonging to the target group^(22)^.

After conducting the narrative interview with nurses, they were encouraged to recommend other nurses who shared similar experiences, generating a successive chain of recommendations. This approach expanded the scope of the research and fostered the establishment of a network of trust, ensuring a safe and anonymous environment. The final number of interviewees was determined by the principle of saturation, the point at which further interviews no longer provided relevant information, indicating repetition and homogenization of responses^(23)^.

The chain sampling process, using the “snowball” method, began with contact and a narrative interview with a black nurse from the institution, selected for fitting the profile established by the study and for playing the role of a key participant (seed). Her position as a key player within the institutional context favored the collection of multiple initial recommendations of potential participants. During this process, two refusals were recorded, resulting in a total of 12 black nurses interviewed.

The research team consisted of eight nurses (faculty and master’s students) who self-identified as black and white, ensuring a multiplicity of backgrounds that contributed to the analysis. The field team, consisting of two master’s students (one black and one white), was also formed in the same vein. The research team had no prior relationship with any of the nurses interviewed.

### Data analysis

The content analysis technique proposed by Bardin^(24)^ was adopted. This empirical and systematic method examines individual and collective narratives, identifying elements that allow inferences about their contexts of narrative content production. Structured in three chronological poles-pre-analysis, material exploration, and results treatment, inference, and interpretation-this approach allows for the rigorous systematization of the empirical *corpus*, enabling the categorization and interpretation of the meanings present in the analyzed material.

In pre-analysis, the empirical material was systematized and selected, following the criteria of exhaustiveness, representativeness, homogeneity, and relevance. During material exploration, the Units of Meaning (UMs) were coded, decomposed, and enumerated: Registration Units (RUs) and Context Units (CUs). RUs were textual segments with relevant semantic value, used as the basis for frequency counting and categorization. CUs were nominations with textual excerpts necessary to understand the meaning of the RUs.

The criterion for enumerating the UMs was the frequency of occurrence of the units, based on the assumption that the recurrence of elements lends greater analytical relevance to the content. Categorization consisted of classifying the recording units according to semantic analogy criteria, enabling the grouping of elements with common characteristics under generic thematic categories, thus conferring cohesion to the data set. Finally, the results treatment, inference, and interpretation stage consisted of producing analyses based on the adopted theoretical framework, linking the empirical categories to the conceptual problematizations that guide the study, presented in this paper in the Discussion section.

Three categories emerged from this process: (1) Open wounds under white coats: intersectionality in black nurses’ journey; (2) Distress that penetrates the skin: the impact of intersectional violence on mental health; and (3) Networks, prayers, and tides: black nurses’ survival strategies. For the sake of in-depth analysis and given the page limit, the second category will be presented in this document.

### Theoretical framework

This qualitative study uses as its theoretical framework the contributions of Lélia Gonzalez^(17)^, Sueli Carneiro^(25)^, and Kimberlé Crenshaw^(26)^, whose theoretical productions are fundamental for understanding the intersectional violence that permeates black nurses’ experiences. Lélia Gonzalez^(17)^ introduces the concept of “cultural neurosis” to describe how racism and sexism intertwine in black women’s daily lives, highlighting the psychological dimensions of these oppressions. Sueli Carneiro^(25)^, In turn, it addresses the “raciality device” as a structural and historical mechanism responsible for ordering social relations through the production and perpetuation of racial hierarchies, especially among racialized people. Kimberlé Crenshaw^(1,26)^, with the concept of “intersectionality”, offers an analytical tool for understanding how racism, sexism, and classism overlap and reinforce each other, revealing the multiple layers of discrimination faced by subalternized groups. The articulation of these authors allows for an analysis that considers the structural and subjective dimensions of oppression, highlighting its manifestations in labor relations and healthcare practices.

## RESULTS

Twelve narrative interviews were analyzed, totaling 480 minutes of testimonies about their experiences in hospital settings and its impact on their mental health. Participant profiles reveal that 75% self-identified as black, and 25% as brown. Seventy-five percent of participants had technical training in nursing prior to graduation. Concerning employment status, 33% held two jobs simultaneously.

In the category analyzed in this study (“Distress that penetrates the skin: the impact of intersectional violence on mental health”), the narratives revealed how intersectional violence affects not only the daily work routine but also the emotional and subjective dimensions of these women, compromising their mental health. As for psychological distress, the interviewees reported daily microaggressions that result in emotional distress. The constant need to adapt and resist violence generates a state of continuous alert and emotional exhaustion, as illustrated in the following excerpts:

*We feel* [racism]*. These are things that I think only black people will feel* [...] *before the speech, there’s the look, and we perceive that look* [...] *like I told you, it doesn’t affect me anymore, but it did in the past. Today, I’m very calm about it. Always in a kind of intrinsic way. Like, speech, gestures, it bothers me, it’s obvious.* (TIYE)

*I don’t arrive there as a trained professional. I arrive there as a black woman, with the training I received in the countryside, and all of this impacts my arrival in that place and how people look at me, too, right?* (KAHINA)

*Sometimes, jokingly. But then we overlook it, right? But there’s a sexism there. Always, because “it could only be a woman”. That kind of thing* [...]. (AKOSUA)

*The department head said, That’s why I like working only with men. Because men don’t menstruate, they don’t cause problems, they don’t have children. I’ve seen that kind of thing here before. From a doctor.* (MENELIK)

*And why is it that when we work in hospitals with medical directors, they hold the power? And who are they? They are, for the most part, white, male, and wealthy. They’re not used to dealing with black people discussing ideas. Black people are the servants in their homes-the drivers, the cleaners, the cooks.* (AMINA)

The reports also highlight the perception of subtle glances and comments, demonstrating that racial and gender prejudice manifests itself daily and is often hidden. The development of resistance mechanisms, while necessary, often occurs over these women’s mental health.

Intersectional psychological distress also manifests as a result of work overload, professional devaluation, and the several demands faced. The narratives reflect that, in addition to symbolic violence, there is institutional pressure that exacerbates emotional distress.

*Work stress* [...] *I have different schedules, one day job, another night job. Sleep is a complicated issue for me because some days I sleep during the day, some days I sleep at night, and some days I don’t sleep at all. For instance, right now I’m on the go, so this makes us more tired and unmotivated* [...] *because I’m a mother, I’m a woman, I’m a healthcare professional, I’m a wife, and I have many roles, but I am one. I’m a nurse here, but I’m not the number xxx. I’m not that number. I’m a nurse, a mother, a nursing technician. In the hospital, I have these multi-roles. I am that person* [...] *I have a little sadness* [...] *is having the need, for instance, to have two jobs in order to have a stable life* [...] *I left the CME because I was harassed, I think I was getting sick, it was a demand, harassment* [...] “*It could only be black to act like that”; “I don’t want to be treated by her”. I’ve been in situations like that, for being a woman, for being a black woman.* (YAA)

*Today, I see that it’s a bit tiring. I don’t know if I would do nursing today, honestly, because of the emotional exhaustion and lack of appreciation. We see that with each passing day we’re not being valued as we should be.* (TIYE)

*One of my technicians was verbally attacked at night and she had a heart attack* [...]*_It’s not something blatant* [racism]*, it’s a veiled attitude. Like, if there are two of us in this room, the person will address you and not me. They don’t recognize me as a managerial authority because of my skin color* [...] *and she can’t see black skin as superior, always subordinate. This happens* [...] *not just here at work, right? Life as usual.* (MAKEDA)

*Oh, I think so-and-so doesn’t want to be assisted by me.* (NERFETITI)

*“No, I don’t want a black woman to change my diaper”. I didn’t even know what to say, I looked at her and said, “No?!” I think the diaper change was the only thing left. Then I left crying, I didn’t answer, I didn’t say anything.* (AMINA)

The excerpts expose the physical and emotional demands faced by black nurses, who must deal with work overload, prejudice, and the invisibility of their professional skills. These findings point to the intersection of race/color, gender, and class as social markers of difference that directly influence black nurses’ mental health in hospital settings.

## DISCUSSION

### Impacts of intersectional violence black nurses’ psychological distress

Intersectionality, as an analytical tool, allows us to understand how race, gender, and class oppressions overlap and intensify their emotional impact on individuals, generating unique psychological distress. Kimberle Crenshaw^(26)^, who coined the term “intersectionality”, states that this concept and its critical applicability are essential to understanding how multiple forms of oppression intersect, giving rise to unique experiences of discrimination and violence for black women. The intersectional perspective highlights that racism, sexism, and classism do not operate in isolation, but in conjunction, amplifying their impacts and intensifying black women’s emotional distress. Crenshaw argues that intersectionality is fundamental to understanding these women’s experiences, who are often marginalized and invisible, not only due to their race or gender alone, but through the interplay of these factors^(26)^.

By prioritizing a single dimension of discrimination, we neglect intersectional violence, resulting in a lack of public policies and actions that fully address black nurses’ needs. Violence, in this context, transcends explicit physical or verbal abuse, also encompassing symbolic and emotional violence, observable in settings of professional devaluation, microaggressions, and exclusionary practices, common in black nurses’ experience.

The black nurses interviewed for this study point to a specific professional profile: the significant presence of black nurses (75%) may be related to the methodological strategy adopted, specifically snowball sampling, in which initial participants (“seeds”) recommend others with similar profiles, influencing the composition of the subsequent group. The predominance of professionals with technical training (75%) reveals a trajectory in which this qualification serves both as a gateway into the health field and as a path for professional mobility. This trajectory may reflect the urgency of entering the job market, often driven by socioeconomic barriers that hinder direct access to higher education. It is noteworthy that 33% of participants hold two jobs, highlighting the work overload that falls on a significant portion of professionals. The most common double-shift configuration involves simultaneously performing the roles of nurse and nursing technician, which points to strategies for supplementing income and diversifying employment in a job market marked by precarious contractual conditions.

The collected testimonies reflect how invisibility and racial subordination, a product of intersecting axes of power, characterize black nurses’ experiences in the workplace, highlighting the weight of the structural inequalities they face. This confrontation is not limited to professional training but extends to the influence of racial identity on how they are perceived and treated in hospital settings. These experiences of marginalization, such as feeling observed differently or devalued, are intensified by the pressure to conform to behavioral and aesthetic standards often distant from their sociocultural reality.

Lélia Gonzalez (1935-1994), a Brazilian anthropologist, philosopher, and activist, developed a fundamental theoretical framework for understanding racial and gender dynamics in Brazilian society. Her concept of “Amefricanity” proposes an epistemology that values the leading role of black women in Brazilian identity construction, challenging the hegemony of Eurocentric thought. In her works, Gonzalez develops a critical analysis of how colonial structures remain active in contemporary society, manifesting themselves through racism and sexism, which combine to marginalize black women^(17)^. Her theoretical contribution allows us to understand how power structures operate in the production of psychological distress among black women, especially in professional settings historically marked by racial and gender hierarchies, such as nursing.

Black women’s psychological distress demands an intersectional analysis that considers the various forms of oppression they experience, such as racism, sexism, and socioeconomic stratification, embodied in classism. The impact of these structural oppressions on black nurses’ mental health manifests itself in their daily work, characterized by both explicit and covert discrimination. Lélia Gonzalez^(17)^, in her research on the interrelationships between race, gender, class, and culture, introduces the concept of cultural neurosis to describe the profound effects of racism and sexism on black women’s subjectivity.

According to the author, Brazilian society is shaped by a veiled racism that disguises itself under the myth of racial democracy, but which, in fact, produces symbolic and emotional violence with serious consequences for black women’s psychological well-being. In the context of nursing, these experiences are intensified by the triple oppression: professional devaluation combined with racial, gender, and class subordination^(17)^.

Gonzalez^(17)^ emphasizes that cultural neurosis internalizes racism and sexism as social norms, leading black women to question their worth and potential, reinforcing feelings of anxiety and insecurity and limiting their self-affirmation. For black nurses, this dynamic is exacerbated by insufficient institutional support and protective policies that recognize the intersectionality of their experiences. Thus, Gonzalez’s theory allows us to understand how racism and sexism, structurally rooted in institutions, generate emotional distress and jeopardize these professionals’ working conditions, perpetuating inequalities that impact their mental health and, consequently, their professional practice.

The deterioration of mental health, stemming from cultural neurosis, exacerbates the psychological distress that emerges from the internalization of these oppressions as social norms. Racism, even when camouflaged under veiled forms of discrimination, is perceived by black women professionals, directly affecting their self-esteem and emotional balance. Resisting this violence requires a continuous adaptive effort that culminates in emotional exhaustion, perpetuating cycles of psychological distress. From an intersectional perspective, the combined weight of racism, sexism, and classism lends these experiences greater depth, complexity, and challenge. These forms of discrimination constitute barriers to well-being and limit the professional advancement of these women. The lack of institutional policies that consider these dimensions widens inequalities and contributes to psychological distress. Therefore, it is imperative to adopt inclusive and conscious measures to preserve mental health and improve these professionals’ working conditions^(17)^.

Sueli Carneiro, a Brazilian philosopher, writer, and anti-racist activist, has established herself as one of the leading thinkers on race relations in the Brazilian context. Her concept of the raciality device refers to a structural and historical mechanism that organizes social relations based on the production and maintenance of racial hierarchies. Inspired by Foucault’s perspective on power devices, the raciality device articulates practices, discourses, and institutions that promote the differentiation, subordination, and control of racialized bodies, especially black people. This concept highlights how racism is not merely an individual or episodic manifestation, but a system of domination that manifests itself in multiple spheres of social life, including work, education, health, and access to justice. In the Brazilian context, Sueli Carneiro highlights that the racialization apparatus operates through the denial and devaluation of black cultures and identities, while simultaneously naturalizing privileges associated with whiteness, reinforcing a logic of exclusion and marginalization^(25)^.

Hence, the author argues that black women are simultaneously affected by structures of racial, gender, and class domination, producing unique experiences that cannot be reduced to just one of these dimensions. Brazilian structural racism operates through mechanisms of exclusion that limit black women’s access to spaces of power and prestige^(25)^. This fact particularly affects nursing, which constitutes a significant workforce. Her studies on epistemicide highlight how the experience, practice, and knowledge produced by black women are systematically devalued and rendered invisible in academic and professional institutions, contributing to the maintenance of racial hierarchies and the production of psychological distress among these women^(25)^.

The structural racism and sexism present in healthcare institutions overwhelm these professionals, not only physically but also emotionally. The struggle for recognition, respect, and dignity, mentioned in the interviews, is a constant in the journey of these nurses. While seeking to affirm their identities and value their skills, they face an environment often unprepared to deal with the intersectional complexity of their experiences.

Workplace violence significantly contributes to emotional distress, particularly for black women, who face daily manifestations of intersecting oppression based on race, gender, and social class. In the nursing context, reports indicate that this violence manifests itself both explicitly, through physical and verbal aggression, and subtly, through microaggressions and institutional discrimination. The convergence of structural racism and sexism creates a hostile work setting that exacerbates these professionals’ emotional distress.

A study by Carvalho *et al*.^(27)^ supports those of the present investigation, stating that “the predominance of women in nursing and the concentration of positions of power occupied by men contribute to the maintenance of relationships of oppression and vulnerability”. In the case of black nurses, this vulnerability is aggravated, as “structural and institutional racism acts as an additional layer of oppression, reinforcing discriminatory practices and hindering access to effective protection mechanisms”, which demonstrates not only the frequency of the phenomenon, but also its naturalization as an inherent part of the daily life of nursing.

From an intersectional perspective, understanding workplace violence among black nurses implies recognizing that gender, race, and profession do not act in isolation, interacting to increase vulnerability^(27)^. This overlapping of structural inequalities restricts the possibilities for reporting and addressing issues, directly affecting professionals’ well-being and quality of care provided.

The debate is also supported by studies focusing on the specific context of the Brazilian Health System, where such violence manifests itself through discrimination, moral and sexual harassment, professional devaluation, and wage inequality, creating hostile work settings for women. As Cordeiro and Diniz^(28)^ point out, these experiences intertwine gender, structural racism, and socioeconomic inequalities, increasing barriers to professional development and compromising the guarantee of decent working conditions.

The authors’ analysis reveals that the combination of gender, race, class, and function accentuates workers’ vulnerability, especially black women, restricting their access to institutional resources, professional recognition, and effective protection mechanisms. This configuration directly impacts the processes of reporting and confronting violence, in addition to affecting the mental health and retention of these professionals in the sector^(28)^.

Given the above, it is clear that black nurses’ psychological distress emerges from a complex web of intersectional oppressions that demand a multidimensional analysis. Racism and sexism, structurally rooted in healthcare institutions, not only compromise the emotional well-being of these professionals but also impact the quality of care provided and the organizational dynamics of healthcare services themselves. The theoretical contributions of scholars such as Lélia Gonzalez, Sueli Carneiro, and Kimberlé Crenshaw offer a valuable conceptual framework for understanding how these oppressions articulate and materialize in these women’s daily lives, challenging reductionist approaches that disregard the specificity of these experiences.

Given this scenario, it is imperative to develop institutional policies and management practices that recognize intersectionality as a fundamental element for promoting equity in the workplace. Addressing black nurses’ psychological distress requires interventions that transcend the individual level, encompassing structural transformations that destabilize historically constructed racial and gender hierarchies inherited for generations. This process demands not only the implementation of affirmative actions and culturally sensitive mental health programs, but also a profound review of the power relations that permeate the fields of nursing and healthcare as a whole. Only from a truly intersectional perspective will it be possible to build more equitable workplaces that recognize and value black nurses’ contributions, fostering their professional development and preserving their psychological integrity.

### Study limitations

This study should be interpreted in light of its limitations. The first concerns the sample size, which, while adequate for the qualitative approach adopted, may limit the multiplicity of experiences and the possibility of generalizing the findings. Furthermore, the use of the snowball sampling strategy, while effective for accessing hard-to-reach populations, may affect the profile of participants, restricting population and narrative diversity, although this does not invalidate the analysis of experiences as part of the affects that shape subjectivities. Another limitation is that the research was conducted at a single university hospital in Rio de Janeiro, which reduces the diversity of experiences produced in the workplace. However, it is recognized that the structural nature of the intersectional violence identified throughout the study preserves the relevance of the findings, as the reported experiences may reflect realities common to other healthcare institutions, as highlighted by the theoretical framework presented in this manuscript.

### Contributions to nursing, health, or public policy

The study contributes to the field of nursing by highlighting how intersectional violence affects black nurses’ mental health in hospital settings. The intersectional approach broadens understanding of the impacts of structural inequalities on these professionals’ daily lives, reinforcing the need for institutional workplace policies. The findings highlight the importance of fostering workplaces that value diversity and ensure adequate psychological support for nursing professionals, especially those belonging to historically marginalized groups.

## FINAL CONSIDERATIONS

The data obtained from black nurses working at a university hospital in the state of Rio de Janeiro (study participants) demonstrate that intersectionality is not merely a theoretical construct, but a concrete reality that structures and permeates their professional experiences. By giving visibility to these experiences and trajectories, this study contributes to strengthening the debate on black women workers’ mental health, pointing to a pressing need for reformulating institutional policies to promote racial and gender equity in the healthcare workplace.

This research highlighted how the phenomena of structural racism, sexism, and classism permeate and profoundly shape black nurses’ professional and subjective trajectories in the university hospital context. The reported acts of violence are not isolated events, but rather constitute elements of a system that operates continuously and often remains invisible, delegitimizing these professionals and hindering their recognition, professional advancement, and psychosocial well-being. Hospital settings, which should be a space for promoting care, reveal themselves as a setting for symbolic and material disputes, where the hegemonic logic of whiteness is perpetuated through systematic exclusions and daily microaggressions that directly impact these workers’ mental health. The psychological distress identified does not present itself as a contingent phenomenon, but rather as a direct manifestation of historical structures of oppression that persist in contemporary institutions.

## Data Availability

The research data are not available.
