# Supplementary material for: Distress that penetrates the skin: the impact of intersectional violence on black female nurses’ mental health
Source: Rev Bras Enferm. 2026 Jun 12;79:e20250263. doi: 10.1590/0034-7167-2025-0263 (PMC13262873; doi:10.1590/0034-7167-2025-0263)
Supplement: Translation

# Translation: Sofrimento que atravessa a pele: impacto das violências interseccionais na saúde mental de enfermeiras negras

**DOI:** 10.1590/0034-7167-2025-0263pt

**Authors:** Isabela de Freitas Bahia Pereira, Tiago Braga do Espírito Santo, Cecília Maria Izidoro Pinto, Eliane Oliveira Andrade Paquiela, Eluana Borges Leitão de Figueiredo, Ricardo de Mattos Russo Rafael, Caroline Silva Batista Alves, Karoline Nascimento Souza

**Affiliations:** I Universidade do Estado do Rio de Janeiro. Rio de Janeiro, Rio de Janeiro, Brasil; II Universidade Federal do Rio de Janeiro. Rio de Janeiro, Rio de Janeiro, Brasil

**Keywords:** Interseccionalidade, Saúde Mental, Enfermagem, Mulheres, Racismo.

## Abstract

**Objetivos:**

analisar interseccionalmente a experiência de enfermeiras negras em hospital universitário do Rio de Janeiro, com ênfase em seus sofrimentos psíquicos.

**Métodos:**

estudo qualitativo com 12 participantes, utilizando entrevistas narrativas e análise de conteúdo de Bardin.

**Resultados:**

a análise de dados possibilitou emergir três categorias. Neste estudo, destacamos a segunda, “Sofrimento que atravessa a pele: o impacto das violências interseccionais na saúde mental”, que evidencia as demandas emocionais, a sobrecarga laboral, as experiências de preconceito e a recorrente desvalorização profissional. O sofrimento psíquico resulta de opressões interseccionais nas instituições de saúde, afetando o bem-estar e a qualidade da assistência. O aporte teórico de Lélia Gonzalez, Sueli Carneiro e Kimberlé Crenshaw permite compreender como essas opressões se articulam no cotidiano dessas profissionais.

**Considerações Finais:**

as violências interseccionais atuam de forma articulada, produzindo exclusões e sofrimento psíquico, o que reforça a urgência por políticas institucionais antirracistas e de igualdade de gênero.

## INTRODUÇÃO

O debate sobre as desigualdades de gênero e os efeitos deletérios do racismo tem ganhado cada vez mais espaço nas agendas de pesquisas e trabalho da área da enfermagem. No entanto, em geral, acabam sendo tratados como formas de opressões isoladas, aspecto que, ainda que importante, desconsidera o efeito do cruzamento desses sistemas de opressão e violências sobre os corpos. Na tentativa de superar essa lacuna, o conceito de interseccionalidade, conforme formulado por Kimberlé Crenshaw^(1)^ e desenvolvido posteriormente por Patricia Hill Collins^(2)^, fornece uma estrutura teórica fundamental para analisar como múltiplos sistemas de opressão operam de forma simultânea e correlacionada.

Kimberlé Crenshaw^(1)^ argumenta que a interseccionalidade vai além da simples soma de opressões, constituindo-se como um paradigma que evidencia como diferentes eixos de poder se entrecruzam, produzindo experiências singulares de discriminação. Patricia Hill Collins e Sirma Bilge^(2)^ complementam essa perspectiva enfatizando que a interseccionalidade é tanto uma ferramenta analítica quanto uma prática crítica que examina como as relações de poder são mantidas e reproduzidas através da interação entre estruturas sociais, representações culturais e processos identitários. Esse adensamento teórico possibilita uma compreensão mais profunda das desigualdades sociais que afetam grupos historicamente alijados da sociedade^(2)^.

Com a junção de duas ou mais categorias que possibilitam entender a produção do sujeito, tal como classe social, gênero, raça/etnia, sexualidade, entre outras, o conceito traz como ideia básica a possibilidade de explicar como normas, valores, ideologias e discursos influenciam as estruturas sociais e a constituição de identidades. Ademais, possibilita adensar a compreensão de como a formação das identidades que, por natureza teórica, estabelecem-se com base nas diferenças afetam a conformação das subjetividades dos sujeitos^(3)^. Nesta direção, a interseccionalidade, como ferramenta analítica para compreensão do sofrimento psíquico, permite compreender como os marcadores sociais da diferença estão associados e são responsáveis por estruturar vidas através de injustiças e desigualdades.

O conceito de raça está relacionado a circunstâncias históricas. Sempre atravessado por conflitos, relações de poder e decisões autoritárias, as relações raciais são marcadas por preconceitos, desigualdades, construções de estereótipos e discriminação. O racismo, culturalmente inserido na sociedade através de práticas conscientes e inconscientes, está objetivamente atrelado à recorrente relação de superioridade do branco sobre o negro e na constante presença do agenciamento da branquitude na conformação dessas relações de poder e opressão^(4)^.

O gênero, por sua vez, constitui-se como uma categoria analítica e política que evidencia como as diferenças sexuais são convertidas em desigualdades sociais, através de processos históricos e culturais. O gênero não se refere apenas à identidade individual, mas a um sistema de relações de poder que organiza hierarquicamente as experiências sociais de homens e mulheres. No contexto do trabalho em saúde, as relações de gênero se manifestam na feminização de profissões como a enfermagem, produzindo uma distribuição desigual de prestígio, autoridade e remuneração^(5)^. Essas relações se complexificam quando interseccionadas com raça e classe, determinando formas específicas de vulnerabilidade e discriminação que afetam particularmente as mulheres negras no ambiente laboral.

Por fim, a classe social se relaciona à forma de estratificar o poder na sociedade a partir do acesso que estes possuem ao capital econômico^(6)^. Sob a perspectiva interseccional, os eixos de poder supracitados se associam, estabelecendo profundo imbricamento entre as relações raciais, de gênero e de classe na estrutura social, atravessando a vida pessoal e coletiva dos indivíduos e grupos. Essas relações são constituídas simultaneamente como práticas de identidade e alteridade, de inclusão e exclusão, e de igualdade e desigualdade.

Nesse sentido, racismo, sexismo e classismo não podem ser compreendidos de maneira isolada, pois eles se articulam historicamente às dinâmicas econômicas e sociais, operando como mecanismos de legitimação da exploração e da hierarquização social. A partir dessa perspectiva, as desigualdades raciais, de sexo e de classe não são esferas distintas, mas expressões entrelaçadas de um mesmo processo histórico de dominação e subordinação moldado pela colonialidade e pelo desenvolvimento do capitalismo. Destarte, a análise interseccional evidencia o seu profundo enraizamento na estrutura social de indivíduos, grupos e classes, sendo moldadas por dinâmicas de diversidade e desigualdade. Essas dinâmicas alimentam continuamente intolerâncias, preconceitos e estereótipos que, embora se transformem historicamente, persistem como dilemas centrais da contemporaneidade.

O cuidado de enfermagem, historicamente exercido por mulheres, constitui-se como um campo de saberes e práticas atravessado por disputas epistemológicas, relações de poder e processos de subalternização. Ehrenreich e English^(7)^ demonstram que, desde as curandeiras e parteiras até a consolidação da enfermagem profissional, o conhecimento empírico e comunitário das mulheres foi deslegitimado pela ascensão da medicina moderna, marcada por uma lógica masculina, científica e institucionalizada. No Brasil, tal processo se articulou a um projeto higienista e disciplinador, que incorporou padrões eurocêntricos e racializados, vinculando a profissionalização da enfermagem à subordinação ao corpo médico e marginalizando práticas populares de cuidado^(8)^.

Estudos como o de Padilha, Nelson e Borenstein^(9)^ evidenciam que a identidade das enfermeiras no Brasil foi construída a partir de trajetórias biográficas permeadas por tensões, resistências e adaptação às estruturas hierárquicas do cuidado. De forma complementar, Magalhães^(10)^ analisa como estereótipos de gênero associados à ideia de cuidado à natureza feminina, à docilidade e à abnegação se mantiveram como elemento estruturante da percepção social e da valorização profissional da enfermagem. Revisões recentes apontam que a produção científica da área ainda reproduz essas narrativas, embora também se observe o fortalecimento de discursos críticos que as contestam^(11)^.

No contexto hospitalar, compreendido como instituição disciplinar que regula corpos, tempos e saberes^(12,13)^, o trabalho da enfermagem oscila entre a autonomia técnico-relacional e a execução subordinada de prescrições médicas. A literatura acerca das relações raciais demonstra que esse processo produz sofrimento não distribuído de forma homogênea, pois marcadores sociais, como gênero, raça e classe, operam como eixos estruturantes de desigualdade, produzindo vulnerabilidades específicas e naturalizando hierarquias no cuidado^(14)^.

As enfermeiras negras enfrentam demandas constantes que as obrigam a demonstrar seu conhecimento e competência. Esse esforço transforma o trabalho diário em um desafio contínuo de provar, por meio de títulos, especializações e experiências, que possuem formação qualificada e merecem ter reconhecimento, mesmo quando suas capacidades se mostram evidentes para os cargos que ocupam. O exercício profissional, assim, torna-se marcado por uma tensão permanente entre habilidade e validação, demonstrando como o racismo estrutural se inscreve nas práticas cotidianas de cuidado e liderança.

Nessa toada, não é tarefa complexa perceber que a produção de desigualdades tende a gerar sofrimento, aqui compreendido como sofrimento psíquico, estabelecido como um fenômeno complexo e multifacetado, caracterizado por sua natureza subjetiva e individual, manifestando-se de maneira ampla e diversa. Esse sofrimento está frequentemente associado a experiências de forte impacto emocional, e pode se expressar por meio de sentimentos como tristeza, frustração, impotência e percepção de incapacidade. A compreensão ampliada da saúde mental, adotada nesta pesquisa, privilegia uma abordagem integral do sujeito que transcende a mera ênfase no diagnóstico psiquiátrico e na classificação nosológica, concentrando-se nos efeitos que as experiências pessoais exercem na vida social e individual. Nesse sentido, o sofrimento psíquico é concebido como uma expressão subjetiva de vivências adversas que afetam a relação do indivíduo com o mundo ao seu redor. Tal perspectiva supera as limitações dos modelos diagnósticos tradicionais, ao priorizar a subjetividade e as necessidades singulares como eixos centrais do cuidado em saúde mental^(15)^.

Considerando os elementos expostos, evidencia-se a necessidade de produzir conhecimento acerca da intersecção presente na existência da enfermeira negra, destacando aquelas que têm como ambiente laboral o espaço hospitalar. O hospital se configura como local de grande inserção da categoria^(16)^, assim como campo histórico de disputa entre categorias e hierarquias reconhecidas socialmente de maneira desnivelada, com ampliações constantemente tensionadas justamente pelas opressões produzidas pelos sistemas de racialização, de iniquidades de gênero e de diferenciação de classes.

Nesse sentido, o estudo, ao dar voz e afirmar essas mulheres como interlocutoras válidas de suas existências, reconhece seus espaços, valorizando suas experiências e vivências no cotidiano no trabalho. Para tanto, tem-se como questão de pesquisa: como os eixos interseccionais de poder influenciam a saúde mental e o sofrimento psíquico das enfermeiras negras?

## OBJETIVOS

Analisar a experiência de enfermeiras negras em hospital universitário do Rio de Janeiro, com foco na reflexão acerca de seus sofrimentos psíquicos.

## MÉTODOS

### Aspectos éticos

O estudo foi conduzido de acordo com as diretrizes éticas nacionais sendo aprovado pelo Comitê de Ética em Pesquisa da Universidade do Estado do Rio de Janeiro (UERJ), cujo parecer está anexado à presente submissão. O Termo de Consentimento Livre e Esclarecido foi obtido de todos os indivíduos por meio escrito, assinado no momento anterior à realização da entrevista narrativa. A participação foi voluntária, e o anonimato das enfermeiras foi garantido por meio da substituição de seus nomes por designações de origem africana, em um gesto simbólico de valorização da ancestralidade e resistência, inspirado nas reflexões de Lélia Gonzalez^(17)^ sobre identidade e cultura.

### Tipo de estudo

Trata-se de pesquisa exploratória de abordagem qualitativa, cuja metodologia busca compreender aspectos da realidade que não podem ser captados por números, fundamentando-se em teorias para investigar fenômenos sociais em profundidade. O estudo tem como objetivo revelar processos ainda pouco conhecidos, sobretudo em relação a grupos específicos, possibilitando tanto a construção de novas perspectivas quanto a revisão e elaboração de conceitos e categorias ao longo do desenvolvimento da investigação^(18)^.

Por sua vez, a pesquisa exploratória busca conhecer e explicar as causas, consequências e características de um fenômeno, promovendo maior conhecimento ao pesquisador sobre a temática. Para tanto, é necessário, no primeiro estágio da investigação, a familiaridade, conhecimento e compreensão, auxiliando no desenvolvimento ou criação de hipóteses explicativas, na determinação de variáveis, na investigação do comportamento humano, e na identificação de conceitos ou variáveis^(19)^.

O estudo seguiu as diretrizes do *COnsolidated criteria for REporting Qualitative research* (COREQ), que estabelece um conjunto de 32 itens de verificação para garantir o rigor metodológico e a transparência na condução e no relato de pesquisas qualitativas. A utilização do COREQ possibilitou uma descrição detalhada e sistemática dos aspectos metodológicos essenciais, com ênfase na reflexividade da equipe de pesquisa, no desenho do estudo e na análise dos dados, contribuindo para a integridade científica da investigação^(20)^.

### Cenário do estudo

A pesquisa foi desenvolvida em hospital universitário do estado do Rio de Janeiro, instituição que atende às exigências do Ministério da Educação e Cultura e que, em sua rotina de trabalho, acolhe estudantes de graduação em enfermagem, pós-graduandos *lato sensu* e *stricto sensu*, além de enfermeiras e profissionais de outras áreas da saúde.

### Fonte de dados

Foram incluídas na amostra mulheres negras (pretas e pardas), com idade acima de 18 anos, formação superior completa em enfermagem e atuação profissional no hospital no momento da coleta. Foram excluídos homens, mulheres autodeclaradas brancas e pessoas fora da prática profissional, afastadas ou em férias.

### Coleta e organização de dados

A coleta de dados foi realizada por meio de entrevistas narrativas^(21)^, método que valoriza a escuta da experiência vivida como conteúdo essencial para análise interseccional. O foco esteve voltado à compreensão das experiências das participantes no ambiente de trabalho hospitalar, especialmente no que se refere ao sofrimento psíquico.

As entrevistas ocorreram durante os meses de julho e agosto de 2024, tendo sido gravadas e transcritas. A equipe de pesquisa proporcionou um ambiente no qual as participantes estivessem confortáveis para compartilhar suas experiências e relembrar o que sentiam, obtendo entrevistas narrativas com duração média de 40 minutos.

Para coleta de dados, adotou-se a estratégia da bola de neve, uma abordagem não probabilística amplamente empregada para acessar populações de difícil acesso, como é o caso das enfermeiras negras, frequentemente invisibilizadas em contextos de pesquisa. Essa técnica se baseia na construção de cadeias de referência, em que participantes iniciais (sementes), selecionadas por conveniência, são convidadas a indicar outras pessoas pertencentes ao grupo de interesse^(22)^.

Após a realização da entrevista narrativa com as sementes, elas foram encorajadas a indicar outras enfermeiras que compartilhassem experiências similares, gerando uma cadeia sucessiva de indicações. A abordagem ampliou o alcance da pesquisa, bem como favoreceu o estabelecimento de uma rede de confiança, assegurando um ambiente seguro e anônimo. A definição do número final de entrevistadas foi guiada pelo princípio da saturação, ou seja, o ponto em que novas entrevistas não acrescentavam informações relevantes, indicando repetição e homogeneização nas respostas^(23)^.

O processo de amostragem em cadeia, na modalidade “bola de neve”, foi iniciado a partir do contato e da realização de entrevista narrativa com uma enfermeira negra da instituição, selecionada por se enquadrar no perfil estabelecido pelo estudo e por desempenhar o papel de participante-chave (semente). Sua posição de referência no contexto institucional favoreceu a obtenção de múltiplas indicações iniciais de potenciais participantes. Ao longo desse processo, registraram-se duas recusas, resultando, ao final, em um total de 12 enfermeiras negras entrevistadas.

A equipe da pesquisa foi composta por oito enfermeiros e enfermeiras (docentes e mestrandas) autoidentificados(as) como pretos(as), negros(as) e brancos(as), assegurando a multiplicidade de trajetórias que contribuem para a análise. No mesmo diapasão, constituiu-se a equipe de campo, composta por duas mestrandas (uma preta e uma branca). A equipe de pesquisa não tinha relação prévia com nenhuma das enfermeiras entrevistadas.

### Análise dos dados

Adotou-se a técnica de análise de conteúdo proposta por Bardin^(24)^, método empírico e sistemático que examina narrativas individuais e coletivas, identificando elementos que possibilitam inferências sobre seus contextos de produção do conteúdo narrativo. Estruturada em três polos cronológicos - pré-análise, exploração do material, e tratamento dos resultados, inferência e interpretação -, essa abordagem permite a rigorosa sistematização do *corpus* empírico, viabilizando a categorização e a interpretação dos sentidos presentes no material analisado.

Na pré-análise, o material empírico foi sistematizado e selecionado, tendo sido obedecidos os critérios de exaustividade, representatividade, homogeneidade e pertinência. Na exploração do material, realizaram-se a codificação, decomposição e enumeração das Unidades de Significado (USs), a saber: Unidades de Registro (URs); e Unidades de Contexto (UCs). As URs foram os segmentos textuais com valor semântico relevante, utilizadas como base para a contagem frequencial e categorização. As UCs foram as nomeações com trechos textuais necessários à compreensão do significado das URs.

O critério para a enumeração das USs foi a frequência de ocorrência das unidades, partindo-se do pressuposto de que a recorrência dos elementos confere maior relevância analítica ao conteúdo. A categorização consistiu na classificação das unidades de registro segundo critérios de analogia semântica, possibilitando o agrupamento de elementos com características comuns sob categorias temáticas genéricas, conferindo coesão ao conjunto de dados. Por fim, a etapa de tratamento dos resultados, inferência e interpretação consistiu na produção de análises fundamentadas no referencial teórico adotado, articulando as categorias empíricas às problematizações conceituais que orientam o estudo, apresentadas neste estudo, na seção Discussão.

Emergiram deste processo três categorias: (1) Feridas abertas sob jalecos brancos: interseccionalidade na travessia das enfermeiras negras; (2) Sofrimento que atravessa a pele: o impacto das violências interseccionais na saúde mental; e (3) Redes, preces e marés: estratégias de sobrevivência da enfermeira negra. Para este documento, em nome do aprofundamento da análise e considerando o limite de laudas, será apresentada a segunda categoria.

### Referencial teórico

O presente estudo qualitativo tem como referencial teórico as contribuições de Lélia Gonzalez^(17)^, Sueli Carneiro^(25)^ e Kimberlé Crenshaw^(26)^, cujas produções teóricas são fundamentais para a compreensão das violências interseccionais que atravessam a experiência de enfermeiras negras. Lélia Gonzalez^(17)^ introduz o conceito de “neurose cultural” para descrever como o racismo e o sexismo se entrelaçam no cotidiano de mulheres negras, destacando as dimensões psíquicas dessas opressões. Sueli Carneiro^(25)^, por sua vez, aborda o “dispositivo de racialidade” como um mecanismo estrutural e histórico responsável por ordenar as relações sociais por meio da produção e perpetuação de hierarquias raciais, em especial das pessoas racializadas. Kimberlé Crenshaw^(1,26)^, com o conceito de “interseccionalidade”, oferece uma ferramenta analítica para compreender como o racismo, o sexismo e o classismo se sobrepõem e se reforçam, revelando as múltiplas camadas de discriminação enfrentadas por grupos subalternizados. A articulação dessas autoras permite uma análise que considera as dimensões estruturais e subjetivas das opressões, evidenciando suas manifestações nas relações de trabalho e nas práticas de cuidado em saúde.

## RESULTADOS

Foram analisadas 12 entrevistas narrativas, totalizando 480 minutos de depoimentos acerca de suas vivências no ambiente hospitalar e o impacto destas em sua saúde mental. O perfil das participantes revela que 75% se autodeclararam pretas, e 25%, pardas. Verificou-se que 75% das participantes possuíam formação técnica em enfermagem anterior à graduação. Quanto ao vínculo empregatício, 33% mantinham dois empregos simultaneamente.

Na categoria analisada no presente estudo (“Sofrimento que atravessa a pele: o impacto das violências interseccionais na saúde mental”), as narrativas revelaram como as violências interseccionais afetam não apenas o cotidiano de trabalho, mas também as dimensões emocionais e subjetivas dessas mulheres, comprometendo sua saúde mental. No que se refere ao sofrimento psíquico, as entrevistadas relataram microagressões cotidianas que resultam em desgaste emocional. A necessidade constante de adaptação e resistência às violências gera um estado de alerta contínuo e exaustão emocional, conforme ilustrado nos trechos a seguir:

*A gente sente* [o racismo]. *São coisas que acho que só o preto vai sentir* [...] *antes da fala, tem o olhar e esse olhar a gente percebe* [...] *como eu te falei, isso hoje não me atinge mais, mas já atingiu no passado. Hoje, eu sou muito tranquila em relação a isso. Sempre de uma forma meio intrínseca. Tipo, falas, gestos, isso incomoda, é óbvio.* (TIYE)

*Eu não chego ali como uma profissional formada. Eu chego ali como uma mulher negra, com a formação que eu tive no interior, e tudo isso impacta na minha chegada naquele lugar e como as pessoas me olham também, né?* (KAHINA)

*Às vezes, de brincadeira. Mas aí a gente releva, né? Mas tem um machismo ali. Sempre, porque “só podia ser mulher mesmo”. Essas coisas* [...]. (AKOSUA)

*O chefe do setor falou assim, por isso que eu gosto de trabalhar só com homens. Porque homem não fica menstruado, não dá trabalho, não tem filho. Esse tipo de coisa eu já vi aqui. Por um médico.* (MENELIK)

*E por que quando a gente se insere em hospitais que têm direção médica, eles são o poder? E quem são eles? São, em grande maioria, brancos, homens, alto poder aquisitivo. Não estão acostumados a lidar com pessoas negras discutindo ideias. Os negros são os serviçais da sua casa, o motorista, faxineiro, cozinheiro.* (AMINA)

Os relatos também destacam a percepção de olhares e comentários sutis, evidenciando que o preconceito racial e de gênero se manifesta de maneira cotidiana e, muitas vezes, velada. A construção de mecanismos de resistência, embora necessária, ocorre frequentemente em detrimento da saúde mental dessas mulheres.

O sofrimento psíquico interseccional também se manifesta em decorrência da sobrecarga de trabalho, da desvalorização profissional e das múltiplas demandas enfrentadas. As narrativas refletem que, além da violência simbólica, há uma pressão institucional que agrava o sofrimento emocional.

*O estresse do trabalho* [...] *como eu tenho horários diferentes, um trabalho de dia, outro trabalho à noite. A questão do sono pra mim é complicado porque tem dia que eu durmo de dia, tem dia que eu durmo à noite, tem dia que eu não durmo. Por exemplo, agora eu tô virada, então, assim, isso deixa a gente mais cansado, mais desmotivada* [...] *porque eu sou mãe, eu sou mulher, eu sou profissional de saúde, eu sou esposa e eu tenho vários papeis, mas eu sou uma. Eu sou enfermeira aqui, mas eu não sou a matrícula xxx. Eu não sou esse número. Eu sou enfermeira, mãe, técnica de enfermagem. No hospital, tenho essas multifunções. Eu sou essa pessoa* [...] *eu tenho um pouco de tristeza* [...] *é ter necessidade por exemplo de ter dois empregos para poder ter uma vida estável* [...] *eu saí da CME porque eu fui assediada, eu acho que eu estava adoecendo, era uma cobrança, assédio* [...] *“Só podia ser preto pra agir dessa maneira”; “Eu não quero ser tratada por ela”. Já tive situações assim, por ser mulher, por ser mulher negra.* (YAA)

*Hoje, eu vejo que é um pouco cansativo. Não sei se hoje eu faria enfermagem, pensando bem sincera, por conta do desgaste emocional e da não valorização. A gente vê que cada dia que passa a gente não está sendo valorizado como deveria.* (TIYE)

*Teve uma técnica minha que foi agredida verbalmente à noite e ela infartou* [...]*_não é uma coisa bem escancarada* [o racismo]*, são atitudes veladas. Tipo, se tiver nós duas aqui nessa sala, a pessoa se dirige a você e não a mim. Ela não me reconhece como uma autoridade gerencial por causa da pele* [...] *e ela não consegue enxergar a pele preta como superior, sempre subalterna. Isso aí acontece* [...] *não só aqui no trabalho, né? A vida de sempre.* (MAKEDA)

*Ah, acho que fulano não tá querendo ser atendido por mim.* (NERFETITI)

*“Não, eu não quero que uma negra troque minha fralda”. Eu não sabia nem o que responder, eu olhei pra ela e falei “não?!”. Acho que a troca de fralda que ficou. Aí eu já saí chorando, não respondi, não falei nada.* (AMINA)

Os trechos expõem as demandas físicas e emocionais enfrentadas pelas enfermeiras negras, que precisam lidar com a sobrecarga de trabalho, o preconceito e a invisibilização de suas competências profissionais. Tais achados apontam para a intersecção da raça/cor, gênero e classe como marcadores sociais da diferença que influenciam diretamente a saúde mental das enfermeiras negras no ambiente hospitalar.

## DISCUSSÃO

### Impactos das violências interseccionais no sofrimento psíquico de enfermeiras negras

A interseccionalidade, enquanto ferramenta analítica, permite compreender como as opressões de raça, gênero e classe se sobrepõem e intensificam o impacto emocional no indivíduo, gerando sofrimento psíquico singular. Kimberle Crenshaw^(26)^, que cunhou o termo “interseccionalidade”, afirma que este conceito e sua aplicabilidade crítica são essenciais para compreender como múltiplas formas de opressão se entrecruzam, originando experiências únicas de discriminação e violência para mulheres negras. A perspectiva interseccional evidencia que racismo, sexismo e classismo não operam isoladamente, mas em conjunção, amplificando reciprocamente seus impactos e intensificando o sofrimento emocional das mulheres negras. Crenshaw sustenta que a interseccionalidade é fundamental para apreender as experiências dessas mulheres, frequentemente marginalizadas e invisibilizadas, não apenas em função de sua raça ou gênero isoladamente, mas pela articulação desses fatores^(26)^.

Ao privilegiar uma única dimensão da discriminação, negligenciamos as violências interseccionais, resultando na ausência de políticas públicas e ações que contemplem integralmente as necessidades das enfermeiras negras. A violência, nesse contexto, transcende o abuso físico ou verbal explícito, abrangendo também a violência simbólica e emocional, observável em cenários de desvalorização profissional, microagressões e práticas excludentes, frequentes na experiência de enfermeiras negras.

As enfermeiras negras entrevistadas para este estudo apontam para um perfil profissional: a expressiva presença de enfermeiras pretas (75%) pode se relacionar à estratégia metodológica adotada, especificamente a amostragem em cadeia do tipo “bola de neve”, na qual as participantes iniciais (“sementes”) indicam outras pessoas com perfis semelhantes, influenciando a composição do grupo subsequente. A predominância de profissionais com formação técnica (75%) revela um percurso em que essa qualificação atua tanto como porta de entrada no campo da saúde quanto como via de mobilidade profissional. Tal trajetória pode refletir a urgência de inserção no mercado de trabalho, frequentemente impulsionada por barreiras socioeconômicas que dificultam o acesso direto ao ensino superior. Ressalta-se que 33% das participantes acumulam dois vínculos empregatícios, evidenciando a sobrecarga laboral que recai sobre uma parcela significativa das profissionais. A configuração mais comum de dupla jornada envolve o exercício simultâneo das funções de enfermeira e técnica de enfermagem, o que aponta para estratégias de complementação de renda e diversificação de inserção em um mercado de trabalho marcado pela precarização das condições contratuais.

Os depoimentos coletados refletem como a invisibilidade e a subordinação racial, produto dos eixos de poder interseccionais, caracterizam a experiência da enfermeira negra no ambiente de trabalho, evidenciando o peso das desigualdades estruturais enfrentadas. Esse enfrentamento não se limita à formação profissional, mas estende-se à influência da identidade racial na forma como são percebidas e tratadas no contexto hospitalar. Essas vivências de marginalização, como sentir-se observada diferenciadamente ou desvalorizada, intensificam-se pela pressão para adequação a padrões comportamentais e estéticos frequentemente distantes de sua realidade sociocultural.

Lélia Gonzalez (1935-1994), antropóloga, filósofa e ativista brasileira, desenvolveu um arcabouço teórico fundamental para a compreensão das dinâmicas raciais e de gênero na sociedade brasileira. Seu conceito de “amefricanidade” propõe uma epistemologia que valoriza o protagonismo das mulheres negras na construção da identidade brasileira, desafiando a hegemonia do pensamento eurocêntrico. Em suas obras, Gonzalez elabora uma análise crítica sobre como a estrutura colonial permanece ativa na sociedade contemporânea, manifestando-se através do racismo e do sexismo, que se articulam para marginalizar as mulheres negras^(17)^. Sua contribuição teórica permite compreender como as estruturas de poder operam na produção de sofrimento psíquico entre as mulheres negras, especialmente em ambientes profissionais historicamente marcados por hierarquias raciais e de gênero, como é o caso da enfermagem.

O sofrimento psíquico das mulheres negras demanda uma análise interseccional que contemple as diversas formas de opressão que as atravessam, como o racismo, o sexismo e a estratificação socioeconômica, materializadas no classismo. O impacto dessas opressões estruturais na saúde mental das enfermeiras negras se manifesta em seu cotidiano laboral, caracterizado por discriminações tanto explícitas quanto veladas. Lélia Gonzalez^(17)^, em suas investigações sobre as interrelações entre raça, gênero, classe e cultura, apresenta o conceito de neurose cultural para descrever os efeitos profundos do racismo e do machismo na subjetividade das mulheres negras.

Segundo a autora, a sociedade brasileira é configurada por um racismo velado que se dissimula sob o mito da democracia racial, mas que, efetivamente, produz violências simbólicas e emocionais com graves consequências para o bem-estar psíquico das mulheres negras. No contexto da enfermagem, essas experiências são intensificadas pela tripla opressão: a desvalorização profissional somada à subordinação racial, de gênero e de classe^(17)^.

Gonzalez^(17)^ enfatiza que a neurose cultural internaliza o racismo e o machismo como normas sociais, conduzindo mulheres negras a questionar seu valor e potencial, o que reforça sentimentos de ansiedade e insegurança, limitando sua autoafirmação. Para as enfermeiras negras, essa dinâmica é agravada pela insuficiência de suporte institucional e políticas de proteção que reconheçam a interseccionalidade de suas vivências. Assim, a teoria de Gonzalez possibilita compreender como o racismo e o sexismo, estruturalmente enraizados nas instituições, geram sofrimento emocional e precarizam as condições laborais dessas profissionais, perpetuando desigualdades que impactam sua saúde mental e, consequentemente, o exercício profissional.

O desgaste da saúde mental, derivado da neurose cultural, acentua o sofrimento psíquico que emerge da internalização dessas opressões como normativas sociais. O racismo, mesmo quando camuflado sob formas veladas de discriminação, é percebido pelas profissionais negras, afetando diretamente sua autoestima e equilíbrio emocional. A resistência a essas violências exige um contínuo esforço adaptativo que culmina em exaustão emocional, perpetuando ciclos de sofrimento psíquico. Na perspectiva interseccional, o peso combinado do racismo, do sexismo e do classismo confere a essas experiências maiores profundidade, complexidade e desafio. Essas formas de discriminação constituem barreiras ao bem-estar e limitam a ascensão profissional dessas mulheres. A ausência de políticas institucionais que considerem essas dimensões amplia as desigualdades e contribui para o sofrimento psicológico. Portanto, é imperativo adotar medidas inclusivas e conscientes para preservar a saúde mental e aprimorar as condições laborais dessas profissionais^(17)^.

Sueli Carneiro, filósofa, escritora e ativista antirracista brasileira, consolidou-se como uma das principais pensadoras das relações raciais no contexto brasileiro. Seu conceito de dispositivo de racialidade se refere a um mecanismo estrutural e histórico que organiza relações sociais com base na produção e manutenção de hierarquias raciais. Inspirado na perspectiva foucaultiana de dispositivos de poder, o dispositivo de racialidade articula práticas, discursos e instituições que promovem a diferenciação, subordinação e controle de corpos racializados, especialmente de pessoas negras. Esse conceito evidencia como o racismo não é apenas uma manifestação individual ou episódica, mas um sistema de dominação que se manifesta em múltiplos âmbitos da vida social, incluindo o trabalho, a educação, a saúde e o acesso à justiça. No contexto brasileiro, Sueli Carneiro destaca que o dispositivo de racialidade opera por meio da negação e desvalorização das culturas e identidades negras, enquanto simultaneamente naturaliza privilégios associados à branquitude, reforçando uma lógica de exclusão e marginalização^(25)^.

Nessa direção, a autora argumenta que as mulheres negras são afetadas simultaneamente por estruturas de dominação racial, de gênero e de classe, produzindo experiências singulares que não podem ser reduzidas a apenas uma dessas dimensões. O racismo estrutural brasileiro opera por meio de mecanismos de exclusão que limitam o acesso das mulheres negras a espaços de poder e prestígio^(25)^. Este fato afeta especialmente a enfermagem, constituindo significativa força de trabalho. Seus estudos sobre epistemicídio evidenciam como a experiência, a prática e o conhecimento produzido por mulheres negras são sistematicamente desvalorizados e invisibilizados nas instituições acadêmicas e profissionais, contribuindo para a manutenção de hierarquias raciais e produção de sofrimento psíquico nessas mulheres^(25)^.

O racismo estrutural e o sexismo presentes nas instituições de saúde sobrecarregam essas profissionais, não apenas fisicamente, mas também emocionalmente. A luta por reconhecimento, respeito e dignidade, mencionada nas entrevistas, apresenta-se como uma constante na trajetória dessas enfermeiras que, ao buscar afirmar suas identidades e valorizar suas competências, enfrentam um ambiente frequentemente despreparado para lidar com a complexidade interseccional de suas experiências.

As violências no ambiente de trabalho contribuem significativamente para o sofrimento emocional, particularmente das mulheres negras, confrontadas cotidianamente com manifestações das opressões interseccionais de raça, gênero e classe social. No contexto da enfermagem, os relatos indicam que essa violência se manifesta tanto explicitamente, por meio de agressões físicas e verbais, quanto sutilmente, através de microagressões e discriminação institucional. A convergência entre racismo estrutural e sexismo configura um ambiente laboral hostil que agrava o sofrimento emocional dessas profissionais.

Estudo realizado por Carvalho *et al*.^(27)^ corrobora os da presente investigação, ao afirmar que “a predominância feminina na enfermagem e a concentração de posições de poder ocupadas por homens contribuem para a manutenção de relações de opressão e vulnerabilidade”. No caso das enfermeiras negras, essa vulnerabilidade se agrava, pois “o racismo estrutural e institucional atua como uma camada adicional de opressão, reforçando práticas discriminatórias e dificultando o acesso a mecanismos efetivos de proteção”, o que demonstra não apenas a frequência do fenômeno, mas também sua naturalização como parte inerente ao cotidiano da profissão.

Sob a ótica da interseccionalidade, compreender a violência no trabalho de enfermeiras negras implica reconhecer que gênero, raça e profissão não atuam isoladamente, interagindo de modo a potencializar as vulnerabilizações^(27)^. Essa sobreposição de desigualdades estruturais restringe as possibilidades de denúncia e enfrentamento, afetando diretamente o bem-estar das profissionais e a qualidade do cuidado prestado.

O debate empreendido é, também, sustentado em estudos que têm como foco o contexto específico do Sistema Único de Saúde, no qual tais violências se expressam por meio de discriminação, assédio moral e sexual, desvalorização profissional, e desigualdade salarial, conformando ambientes laborais hostis às mulheres. Conforme apontam Cordeiro e Diniz^(28)^, essas experiências articulam gênero, racismo estrutural e desigualdades socioeconômicas, ampliando as barreiras para o desenvolvimento profissional e comprometendo a garantia de condições de trabalho dignas.

A análise das autoras revela que a combinação de gênero, raça, classe e função acentua a vulnerabilidade das trabalhadoras, especialmente das mulheres negras, restringindo o acesso a recursos institucionais, reconhecimento profissional e mecanismos efetivos de proteção. Tal configuração impacta diretamente os processos de denúncia e enfrentamento da violência, além de afetar a saúde mental e a permanência dessas profissionais no setor^(28)^.

Diante do exposto, é possível perceber que o sofrimento psíquico das enfermeiras negras emerge de uma complexa trama de opressões interseccionais que demandam uma análise multidimensional. Racismo e sexismo, estruturalmente arraigados nas instituições de saúde, não apenas comprometem o bem-estar emocional dessas profissionais, mas também impactam a qualidade da assistência prestada e a própria dinâmica organizacional dos serviços de saúde. As contribuições teóricas de intelectuais como Lélia Gonzalez, Sueli Carneiro e Kimberlé Crenshaw oferecem um arcabouço conceitual valioso para compreender como essas opressões se articulam e materializam no cotidiano dessas mulheres, desafiando abordagens reducionistas que desconsideram a especificidade dessas experiências.

Diante desse cenário, torna-se imperativo o desenvolvimento de políticas institucionais e práticas de gestão que reconheçam a interseccionalidade como elemento fundamental para a promoção da equidade no ambiente de trabalho. O enfrentamento do sofrimento psíquico das enfermeiras negras requer intervenções que transcendam o nível individual, contemplando transformações estruturais que desestabilizem hierarquias raciais e de gênero historicamente constituídas e herdadas por gerações. Esse processo demanda não apenas a implementação de ações afirmativas e programas de saúde mental culturalmente sensíveis, mas também uma profunda revisão das relações de poder que permeiam os campos da enfermagem e da saúde como um todo. Somente a partir de uma perspectiva verdadeiramente interseccional será possível construir ambientes laborais mais equitativos que reconheçam e valorizem a contribuição das enfermeiras negras, fomentando seu desenvolvimento profissional e preservando sua integridade psíquica.

### Limitações do estudo

O presente estudo deve ser interpretado à luz de suas limitações. A primeira diz respeito ao tamanho da amostra, que, embora adequado para a abordagem qualitativa adotada, pode limitar a multiplicidade de experiências e possibilidade de generalização dos achados. Ademais, a utilização da estratégia de amostragem por bola de neve, embora eficaz para acessar populações de difícil acesso, pode afetar o perfil das participantes, restringindo a diversidade populacional e de narrativas, ainda que isso não invalide a análise das experiências como parte dos afetos que conformam as subjetividades. Outra limitação refere-se ao fato de a pesquisa ter sido conduzida em um único hospital universitário do Rio de Janeiro, o que reduz a diversidade de experiências produzidas no ambiente laboral. No entanto, reconhece-se que a natureza estrutural das violências interseccionais identificadas ao longo do estudo preserva a relevância dos achados na medida em que as experiências relatadas podem refletir realidades comuns a outras instituições de saúde, bem como apontado pelo arcabouço teórico apresentado neste manuscrito.

### Contribuições para as áreas da enfermagem, saúde ou políticas públicas

O estudo contribui para a área da enfermagem, evidenciando como as violências interseccionais afetam a saúde mental de enfermeiras negras em ambiente hospitalar. A abordagem interseccional amplia a compreensão dos impactos das desigualdades estruturais no cotidiano dessas profissionais, reforçando a necessidade de políticas institucionais dos ambientes de trabalho. Os achados ressaltam a importância de promover ambientes laborais que valorizem a diversidade e garantam suporte psicológico adequado às profissionais de enfermagem, especialmente àquelas pertencentes a grupos historicamente marginalizados.

## CONSIDERAÇÕES FINAIS

Os dados obtidos junto às enfermeiras negras atuantes em hospital universitário localizado no estado do Rio de Janeiro (participantes do estudo) demonstram que a interseccionalidade não se configura apenas como construto teórico, mas como realidade concreta que estrutura e permeia suas vivências profissionais. Este estudo, ao conferir visibilidade a tais experiências e trajetórias, contribui para o fortalecimento do debate acerca da saúde mental de trabalhadoras negras, apontando para um horizonte premente de reformulação das políticas institucionais, com vistas à promoção da equidade racial e de gênero nos ambientes laborais do setor saúde.

Esta pesquisa evidenciou como os fenômenos do racismo estrutural, do sexismo e do classismo permeiam e configuram profundamente as trajetórias profissionais e subjetivas das enfermeiras negras no contexto hospitalar universitário. As violências relatadas não se caracterizam como eventos isolados, mas constituem elementos de um sistema que opera de forma contínua e frequentemente invisibilizada, deslegitimando essas profissionais e obstaculizando seu reconhecimento, ascensão funcional e bem-estar psicossocial. O ambiente hospitalar, que deveria ser espaço de promoção do cuidado, revela-se como cenário de disputas simbólicas e materiais, onde a lógica hegemônica da branquitude se perpetua por meio de exclusões sistemáticas e microagressões cotidianas que impactam diretamente a saúde mental dessas trabalhadoras. O sofrimento psíquico identificado não se apresenta como fenômeno contingente, mas como manifestação direta de estruturas históricas de opressão que persistem nas instituições contemporâneas.

## Data Availability

Os dados de pesquisa não estão disponíveis.
